# P-60 - THE ‘SUPER FLU’ THAT WASN’T: THE IMPACT OF THE H3N2 SUBCLADE K ON THE SCOTTISH POPULATION, 2025/26

**DOI:** 10.1093/pubmed/fdag046.089

**Published:** 2026-07-09

**Authors:** Mr Ebrahim Makhoul, Dr Stefanie Wilson, Dr Masoumeh Rezaie, Mr Allan Mcleod, Dr Kirsty Morrison, Mr Safraj Shahul Hameed, Mr John Wood, Prof Rory Gunson, Dr Kimberly Marsh

**Affiliations:** Public Health Scotland; Public Health Scotland; Public Health Scotland; Public Health Scotland; Public Health Scotland; Public Health Scotland; Public Health Scotland; West of Scotland Specialist Virology Centre; Public Health Scotland

## Abstract

**Background:**

Seasonal influenza remains unpredictable due to continual viral evolution, particularly within A(H3N2) lineages. Novel subclades can alter transmission, severity, and vaccine effectiveness (VE), underscoring the importance of pathogen-specific surveillance. In 2025/26, Scotland and other European countries identified a new H3N2 subclade, speculatively labelled ’Super Flu’, prompting intensified monitoring.

**Objectives:**

To describe the impact of a drifted influenza A(H3N2) subclade K virus on the Scottish population over the 2025/26 winter season.

**Methods:**

Epidemiological data including laboratory confirmed tests, hospitalisations and mortality were used to quantify severity and transmissibility. Vaccine effectiveness was estimated and compared to previous seasons (methods published elsewhere).

**Results:**

Activity in 2025/26 peaked earlier (week 49, 2,406 cases) compared to the previous season (week 52, 2,954 cases). 73% of detections were influenza A, not subtyped due to resource limitations. Among subtyped influenza A and influenza B specimens, H3N2 dominated in 2025/2026 (86.2%), whereas H1N1 and B co-circulated last season (43.6% and 47.3%, respectively). Through genomic sequencing, subclade K was first identified in week 34 and became the dominant strain by week 39, accounting for 93.2% of all sequenced H3 samples and 71.6% of all influenza sequences. A higher burden was observed in children (<15 years). Early Scottish estimates of VE against influenza hospitalisations were 34% overall (95% CI: 28–39%), consistent with previous seasons, with higher VE in children (61%; 95% CI: 41–74). Hospitalisations peaked at 1,054 in week 49, below the 2024/25 peak of 1,600. Influenza-attributed deaths were 466 in weeks 40-12, compared with 599 in 2024/25.

**Conclusions:**

In 2025/26, H3N2 subclade K emerged early and rapidly dominated, affecting children more, possibly due to lower prior H3N2 exposure or pre-vaccination circulation. There was no evidence of increased severity or reduced vaccine effectiveness. Subclade K reshaped the season’s timing, not its severity, dispelling early ‘Super Flu’ concerns.

